# Supplementary material for: Widespread losses of pollinating insects in Britain
Source: Nat Commun. 2019 Mar 26;10:1018. doi: 10.1038/s41467-019-08974-9 (PMC6435717; doi:10.1038/s41467-019-08974-9)
Supplement: Supplementary file 1 — Supplementary Information [file 41467_2019_8974_MOESM1_ESM.pdf]

Supplementary Information:

Widespread losses of pollinating insects in Britain

Powney et al.

Supplementary Figure 1 The annual growth rate in occupancy between 1980 and 2013 highlights the variation in trend estimates among species (bees – red, hoverflies – blue). Individual species trends were summarised using the median (points) and 95% credible intervals (bars). Species (n = 4) with either exceptionally large uncertainty (95% credible intervals span greater than 20 percent), or with extremely strong trend estimates (annual trend estimate greater than 10%, or less than -10%) were excluded from this plot.

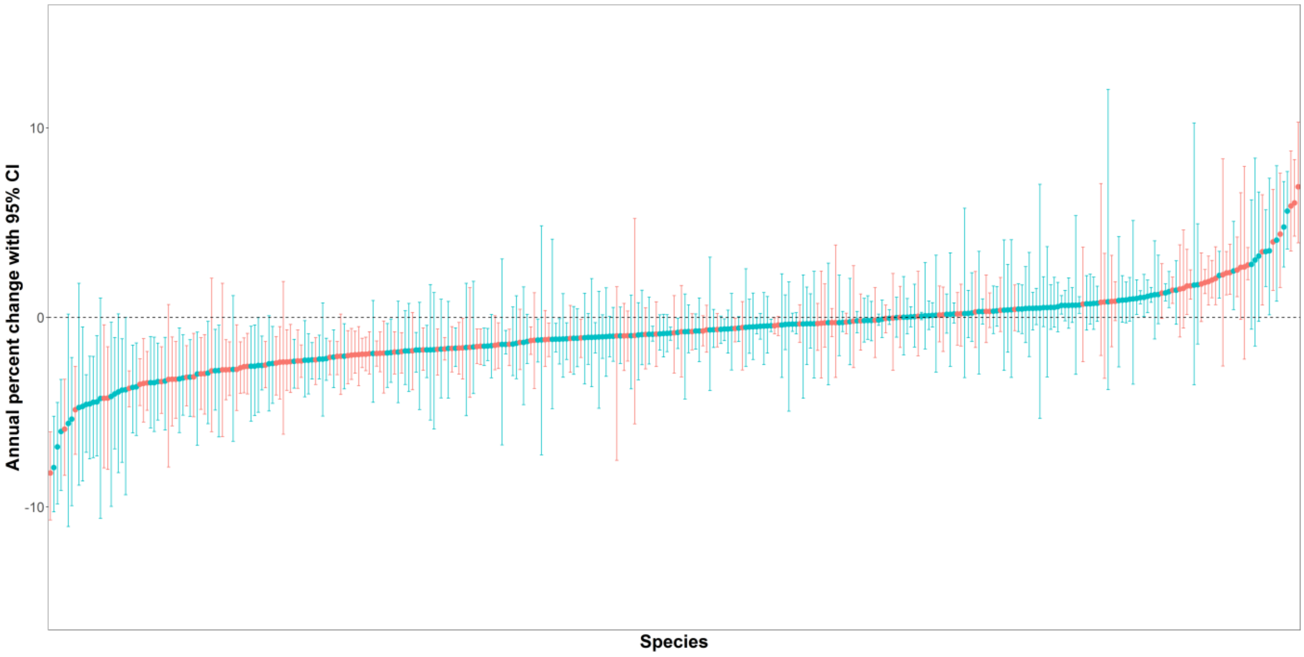

Supplementary Figure 2 First derivatives of average occupancy for a) bees and b) hoverflies. Points are the median estimate of the posterior distribution, with uncertainty presented as the limits of the credible intervals (thin = 95% CI, thick = 80% CI).

a)

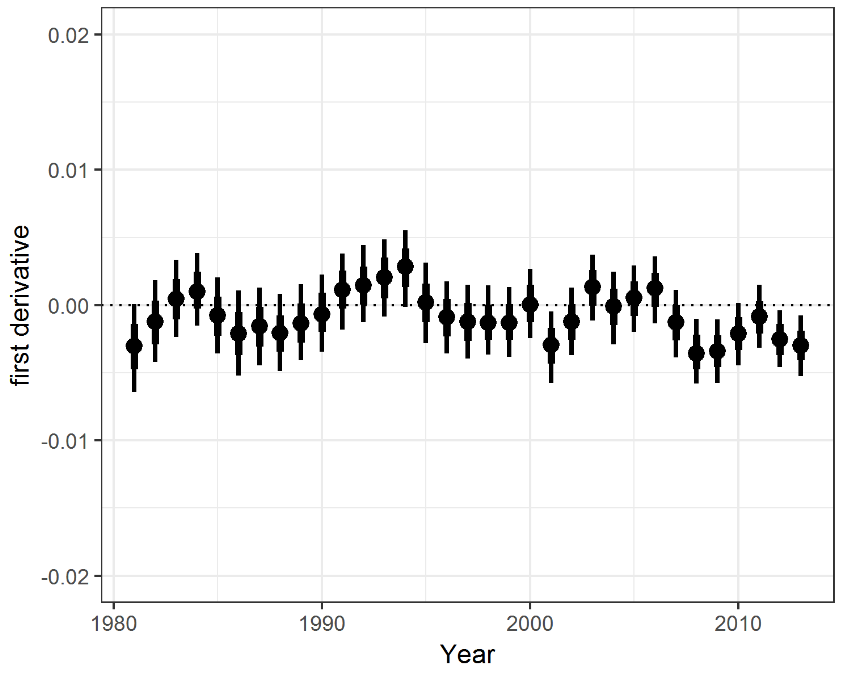

b)

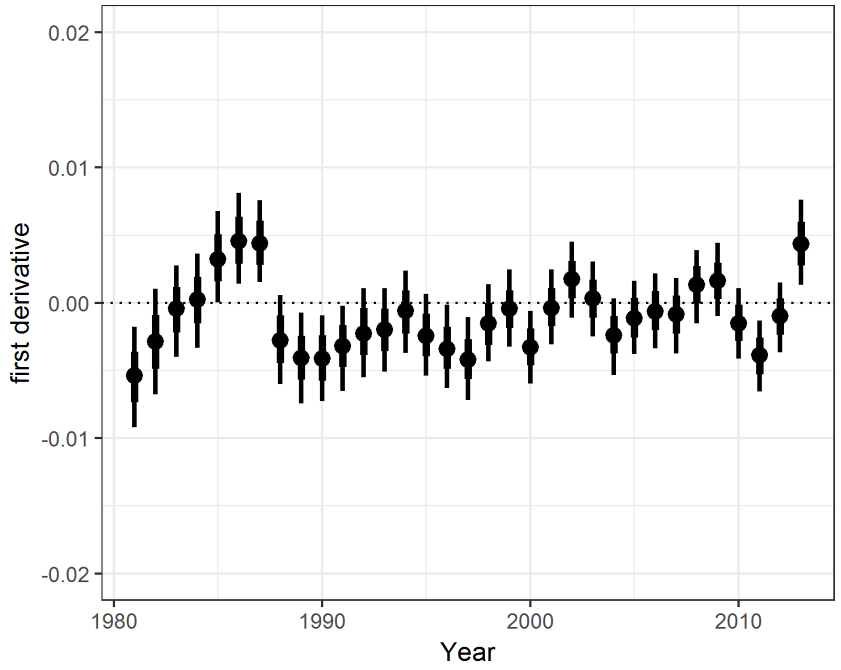

Supplementary Figure 3 Contrasting patterns of change among dominant wild bee crop pollinators (n = 22 species, grey) and other bee species (n = 117 species, green). Trend lines show average occupancy across species within each group, calculated as the geometric mean annual occupancy, dashed lines delimit the 95% credible intervals.

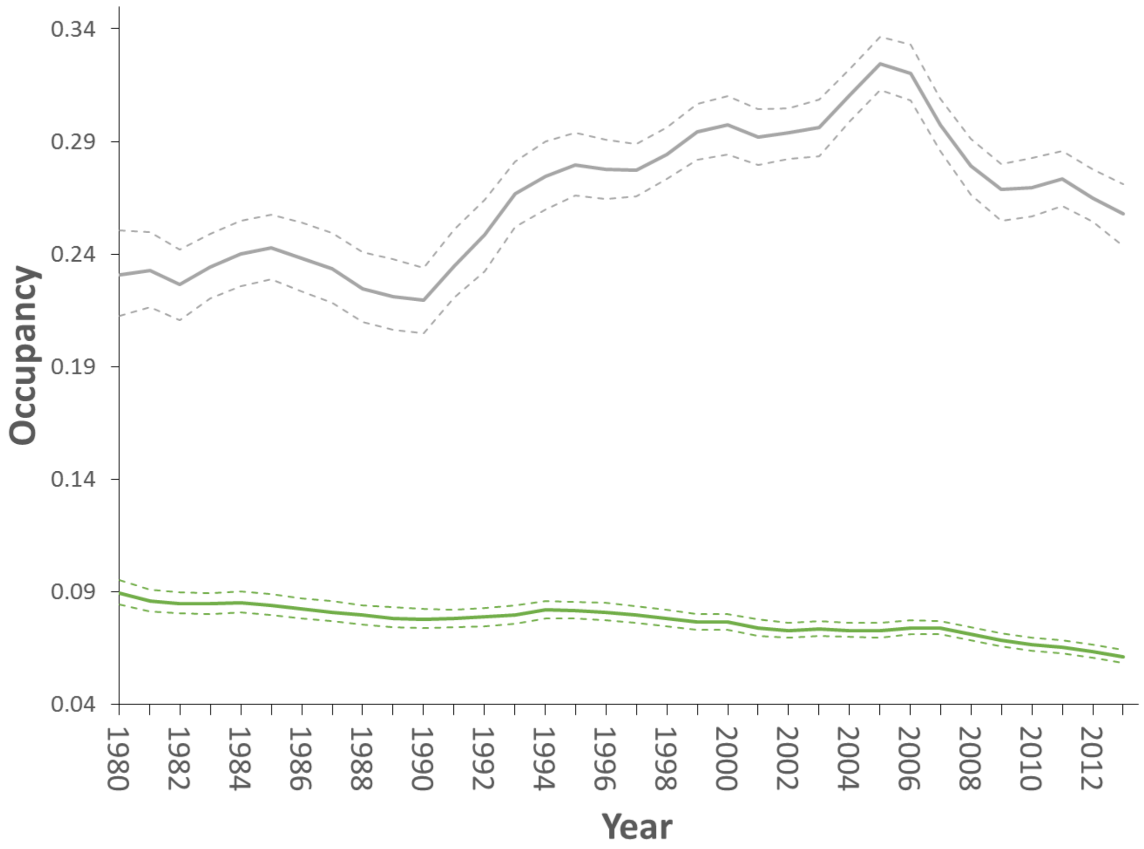

Supplementary Figure 4 Contrasting patterns of change among eusocial (n = 21 species, red) and solitary (n = 112 species, blue) bee species. Trend lines show average occupancy across species within each group, calculated as the geometric mean annual occupancy, dashed lines delimits the 95% credible intervals.

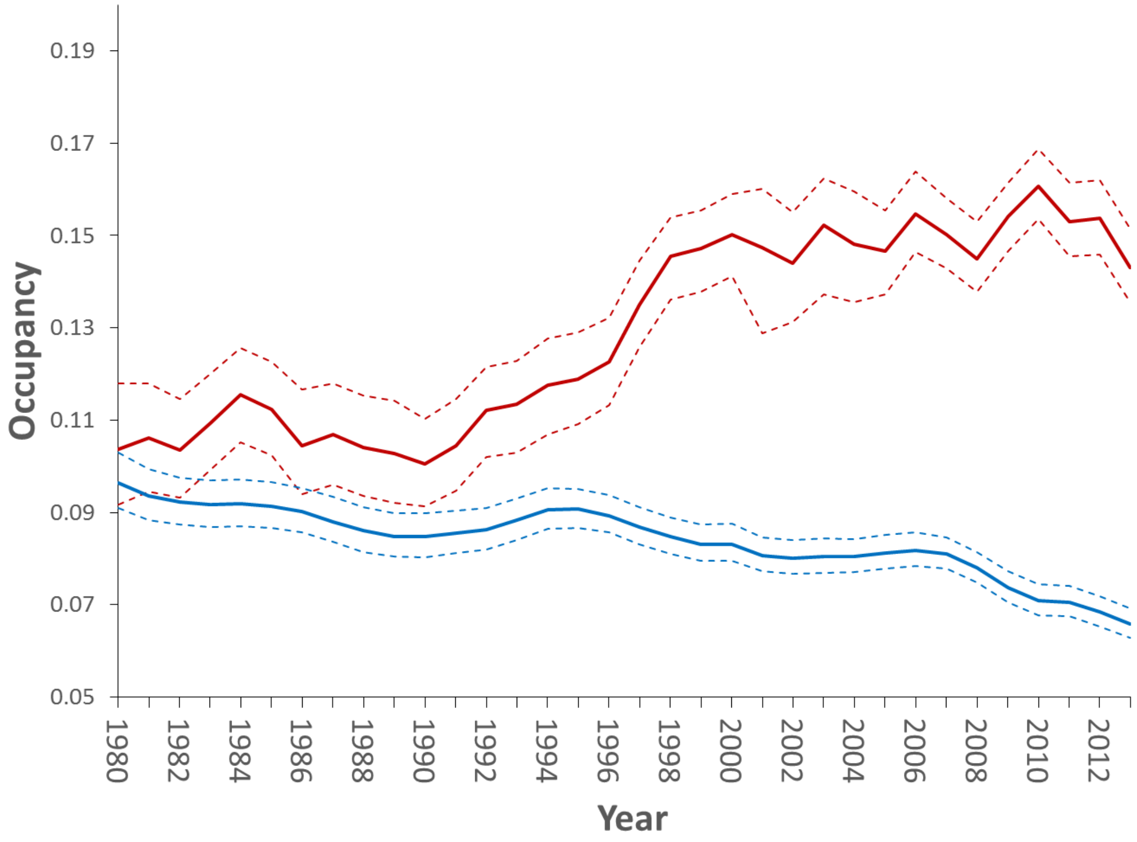

Supplementary Figure 5 Contrasting patterns of change among upland (n = 38 species, red), southern (n = 117 species, grey), widespread southern (n = 107, blue) and widespread pollinators species (n = 149 species, yellow). Trend lines show average occupancy across species within each group, calculated as the geometric mean annual occupancy, dashed lines delimits the 95% credible intervals.

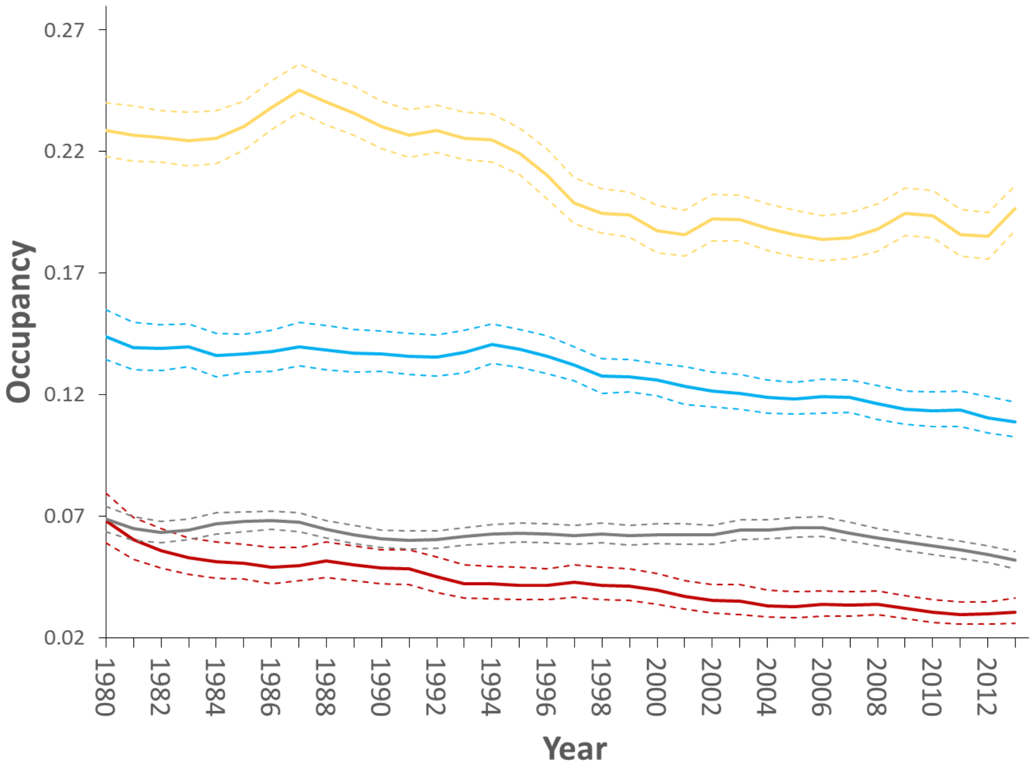

Supplementary Figure 6 Species richness maps based on records collected between 1980 and 2013 at the 10km grid square scale. Each of the four species clusters derived via CLUSTASPEC are presented: a) upland cluster, b) southern cluster, c) widespread southern cluster, and d) widespread cluster. A list of species within each cluster can be found in Supplementary Table 2

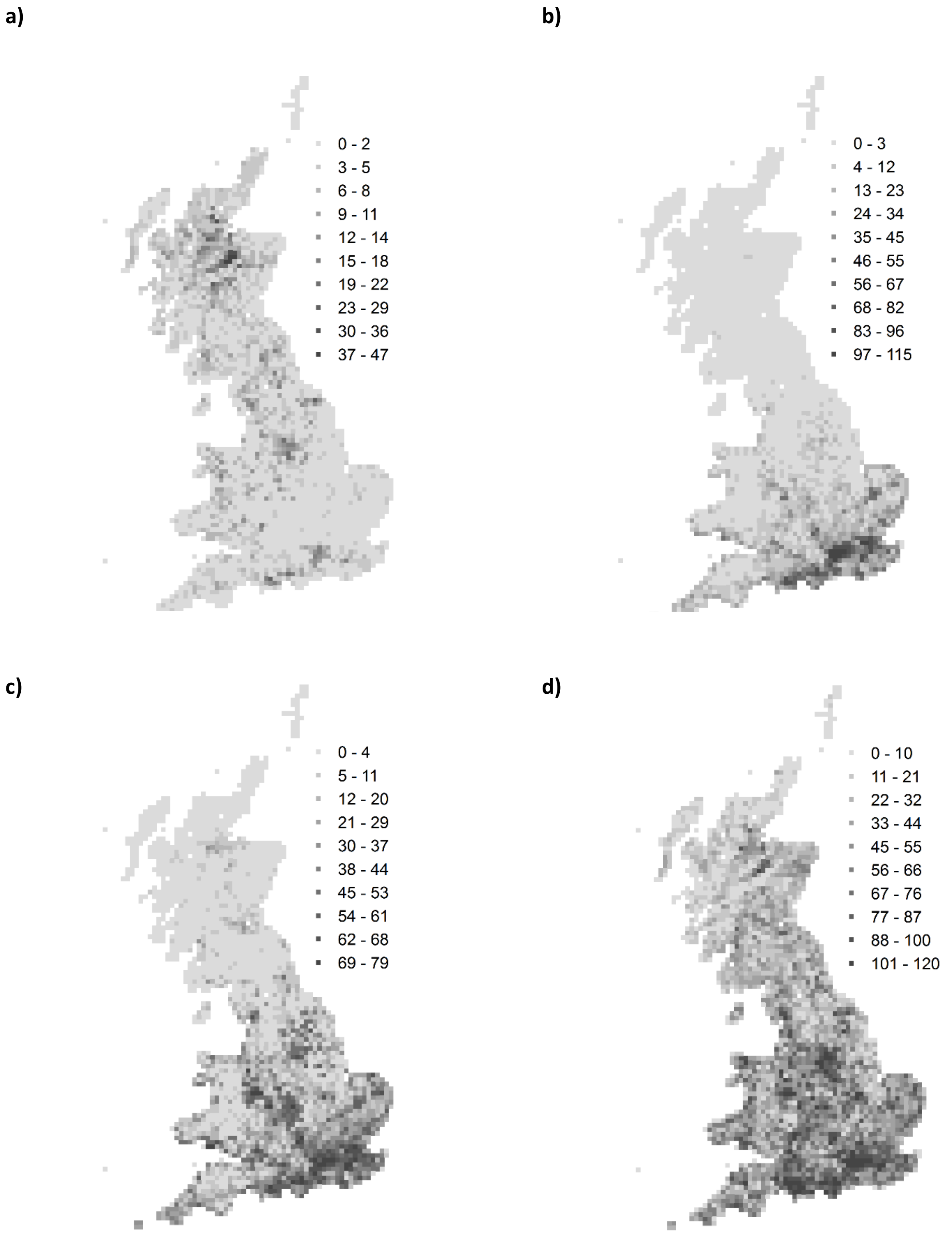

Supplementary Table 1 The percentage of species in each trend category based on a 95% level of certainty. Species were classified as having no clear trend, if the 95% credible intervals of the trend spanned zero.

	**Taxonomic group**		
**Trend**	**Pollinator**	**Bee**	**Hoverfly**
Increasing	11	14	8
No clear trend	56	44	65
Declining	33	42	27
