# Supplementary material for: Widespread losses of pollinating insects in Britain
Source: Nat Commun. 2019 Mar 26;10:1018. doi: 10.1038/s41467-019-08974-9 (PMC6435717; doi:10.1038/s41467-019-08974-9)
Supplement: Supplementary file 3 — Description of Additional Supplementary Files [file 41467_2019_8974_MOESM3_ESM.pdf]

Description of Additional Supplementary Files

File Name: Supplementary Data 1

Description: A summary table showing the breakdown of bee and hoverfly species in each of the trait grouping categories. Non-eusocial species are referred to a solitary species in the text describing this study.
